# Anticancer potential of cryptotanshinone on breast cancer treatment; A narrative review

**DOI:** 10.3389/fphar.2022.979634

**Published:** 2022-09-16

**Authors:** Davood Dalil, Saeid Iranzadeh, Soroush Kohansal

**Affiliations:** Student Research Committee, Faculty of Medicine, Shahed University, Tehran, Iran

**Keywords:** salvia miltiorrhiza, cryptotanshinon, tanshinone C, breast cancer, molecular mechanism, drug combination, estrogen receptor

## Abstract

Breast cancer has recently been known as the first lethal malignancy in women worldwide. Despite the existing treatments that have improved the patients’ prognosis, some types of breast cancer are serious challenges to treat. Therefore, efforts are underway to provide more efficient therapy. Cryptotanshinone (CPT) is a liposoluble diterpenoid derivation of a traditional Chinese herbal medicine called Salvia miltiorrhiza Bunge. It has been considered in the past decades due to its vast therapeutic properties, including anti-tumor, anti-inflammatory, and anti-fibrosis. Recently, studies have found that CPT showed a significant anti-breast cancer effect *in vivo* and *in vitro* through different physiological and immunological mechanisms. This study summarized the latest research findings on the antitumor effect of CPT in breast cancer. Further, the main molecular mechanisms based on breast cancer types and combination with other drugs were reviewed to provide essential evidence for future longitudinal research and its clinical application in breast cancer treatment.

## Introduction

Breast cancer (BRCA) is one of women’s most commonly diagnosed malignancies worldwide. The incidence of BRCA has increased gradually in recent years, becoming the top rank in 2021. (Ma and Jemal, 2013; DeSantis et al., 2014; Harbeck and Gnant, 2017; Sung et al., 2021). Based on evaluation of different biomarkers, including presence of hormone receptors (HR) [such as estrogen receptor (ER)] and overexpression of human epidermal growth factor receptor 2 (HER2), BRCA is divided into four main molecular subtypes: HR+/HER2-, HR+/HER2+, HER2+ and triple negative (TNBC) (Loibl et al., 2021). HR positive BRCA is less malignant than other subtypes. Although there are various BRCA risk factors related to lifestyle (Brody et al., 2007; Kaiser, 2013), medical condition (Anothaisintawee et al., 2013), carcinogenic genes (Gage et al., 2012), etc., it is well known that estrogen and ERs play a pivotal role in the initiation, development, and progression of breast cancer (Platet et al., 2004; Yager and Davidson, 2006). Three types of ER have been identified in BRCA cells, ERα and ERβ, and a G-protein coupled estrogen receptor (GPER) (Girgert et al., 2019).

The successful medical treatments for breast cancer, including surgery, chemotherapy, and radiation therapy, have been associated with better prognosis, causing a dramatic increase in the survival rate of BRCA patients (Abderrahman and Jordan, 2018). However, in some subtypes of BRCA the prognosis is poor (Tong et al., 2018). Success in controlling the progression and treatment of breast cancer with chemotherapy drugs such as tamoxifen and anti-estrogens depends on the presence of ERs, especially ERα. Thus, the treatment of ERα-negative BRCA, accounting for 40% of BRCAs, is challenging. A subgroup of ERα- breast cancer overexpress the HER2, and there are few drugs for its treatment. Another group is TNBC which is so malignant to be treated, resulting in a poor prognosis (Tong et al., 2018; Girgert et al., 2019). Also, treatment obstacles, such as multidrug resistance (MDR), decrease the clinical efficacy of treatment in BRCA patients (Merikhian et al., 2017).

Therefore, more studies have been conducted to explore an effective therapeutic agent to improve the prognosis of different subtypes of BRCA, especially by targeting estrogen signaling (Li et al., 2015). Herbal products are a treasure for pharmaceutical development, providing novel biological and natural compounds to develop new medications (Cragg and Newman, 2009). These drugs are known as valuable and safe resources in the treatment of various diseases due to their low price, low adverse effects, and high public availability (Balaña-Fouce et al., 1998).

Cryptotanshinone (CPT) is a liposoluble diterpenoid derivation, that mainly exists in plants of the genus Salvia, including Salvia przewalskii Maxim, Salvia tebesana Bunge., *S. miltiorrhiza* Bunge., among which *S. miltiorrhiza* Bunge, well-known as Danshen, has rich contents of diterpenes (Wu et al., 2020). Recently, CPT has been considered due to its vast range of therapeutic properties, including anti-tumor effects (Jiang et al., 2017), anti-inflammatory (Tang et al., 2011), anti-fibrosis, etc. (Wu et al., 2020). For anti-tumor activity, CPT inhibited the growth of various kinds of tumor cells, including lung cancer (Lee et al., 2008), prostate cancer (Shin et al., 2009), cervical cancer (Ye et al., 2010), leukemia (Kim et al., 2011), and breast cancer (Park et al., 2012). CPT, besides its cytotoxic effect, could prevent cancer cell proliferation and increase anti-tumor immunity simultaneously (Han et al., 2019). Therefore, this study summarized the latest research findings on the anti-breast cancer activity of CPT. Furthermore, the main molecular mechanisms based on breast cancer subtypes and combination with other drugs were reviewed to provide essential evidence for future longitudinal research and possible CPT clinical application in breast cancer treatment.

## Results

The crucial characteristic of an ideal anti-tumor drug is the fewest cytotoxic effect on normal cells while the most cytotoxicity on cancer cells. Studies have demonstrated that CPT has such an anti-tumor effect (Zhang et al., 2018). Regarding anti-breast cancer treatment, CPT could affect different breast cancer cell lines through various mechanisms (Table 1). Zhou et al. (2020) showed that CPT has dose-dependent cytotoxicity on ERα-positive BRCA cells (MCF-7 cells) and ERα-negative BRCA cells (MDA-MB-231), decreasing the survival and proliferation of cancerous cells. Li et al. (2021) showed that CPT in any concentration inhibits the rate of proliferation time/concentration-dependent in MCF-7 and MDA-MB-231 BRCA cells. Also, their experiments on transwell invasion and cell migration demonstrated that MCF-7 cells are more sensitive to CPT than MDA-MB-231 cells. CPT inhibited the invasive ability of BRCA cells in a dose-dependent manner. At the same concentration of CPT, the migration distance of MCF-7 cells is lesser than MDA-MB-231 cells; additionally, increasing the concentration of CPT results in more potent inhibition in the cells migration.

**TABLE 1:** The anti-breast cancer effects of CPT in estrogen-receptor dependent or independent manner.

Author	Estrogen receptor	Breast cancer cell line	CPT inhibiting effect	CPT inducing effect	Main mechanism
Zhou et al. (2020)	Positive	MCF-7	Glycolysis, Cell proliferation, Cell migration, Cell invasion	—	Downregulation of the PKM2/β-catenin axis
	Negative	MDA-MB-231			
Park et al. (2012)	Positive	MCF-7	Cell viability	Apoptosis, Cell sensitivity to chemotherapy drugs	Inducing ER stress-mediated apoptosis through generating reactive oxygen species
Li et al. (2015)	Positive	ZR-75-1, MCF7, MDA-MB-231, MDA-MB-435	ERα-mediated transcriptional activity, Cell growth, Cell survival, Cell proliferation, *In vivo* tumor growth	—	Competitive binding to Estrogen receptor
	Negative				
Pan et al. (2017)	Positive	MCF-7, T47D, MCF-7/ADR, MDA-MB-231, MDA-MB-435	Cell proliferation, Cell viability, *In vivo* tumor growth	Cell sensitivity to the Tamoxifen	Downregulation of ERa-dependent IGF-1/AKT/mTOR pathway
	Negative				
Li et al. (2021)	Positive	MCF-7	Cell viability, Cell proliferation, Cell invasion, Cell migration	—	Reducing CCNA2 and CDK1 expression
	Negative	MDA-MB-231			
Shi et al. (2020)	Negative	SKBR-3	Cell viability, Cell proliferation	Cell cycle arrest	Downregulated GPER mediated PI3K/AKT signaling pathway, Regulated the expression levels of cell cycle-associated proteins
Liu et al. (2016)	Negative	Bcap37	Cell proliferation, Cell migration	Cell apoptosis	Inducing mitochondria-derived ROS/FOXO1 pathway
Liu et al. (2020)	Negative	4T1	Breast cancer lung metastasis, cell invasion, Cell migration, *In vivo* tumor growth	—	Increased bioavailability of nanoparticles co-loaded with silibinin and CPT
Zhang et al. (2015)	Positive	MCF-7	Cell viability	Cell apoptosis	Induction of endoplasmic reticulum stress after exposure to CPT combined with arsenic species
Ni et al. (2021)	Positive	MCF-7	Efflux function of BCRP, Export of chemotherapy drugs from the cancer cells	Cell sensitivity to chemotherapy drugs	Inhibiting oligomer formation of BCRP on the cancer cell membrane
	Negative	MDA-MB-231			
		MCF-7/ADR			

### Cryptotanshinone against estrogen receptor alpha-positive breast cancer cells (MCF-7 cell line)

ERα is a ligand-regulated transcription factor that binds to the estrogen hormone and activates a pathway, which triggers the transcription of ER target genes via binding to the estrogen-responsive elements (EREs) on their gene promoters (Klinge, 2001; Osborne et al., 2001; Deroo and Korach, 2006). Around 70%–75% of BRCAs express ERα in their cells, known as estrogen receptor alpha-positive breast cancer (ERα-positive BRCA) (Cleator et al., 2009; Johnston, 2010) Cryptotanshinone, a homogeneous chemical structure with estrogen, inhibited cell viability and proliferation in ERα-positive cells more effectively than in ERα-negative cells in a dose-dependent manner (Gong et al., 2012; Li et al., 2015; Pan et al., 2017; Li et al., 2021).

**FIGURE 1:**
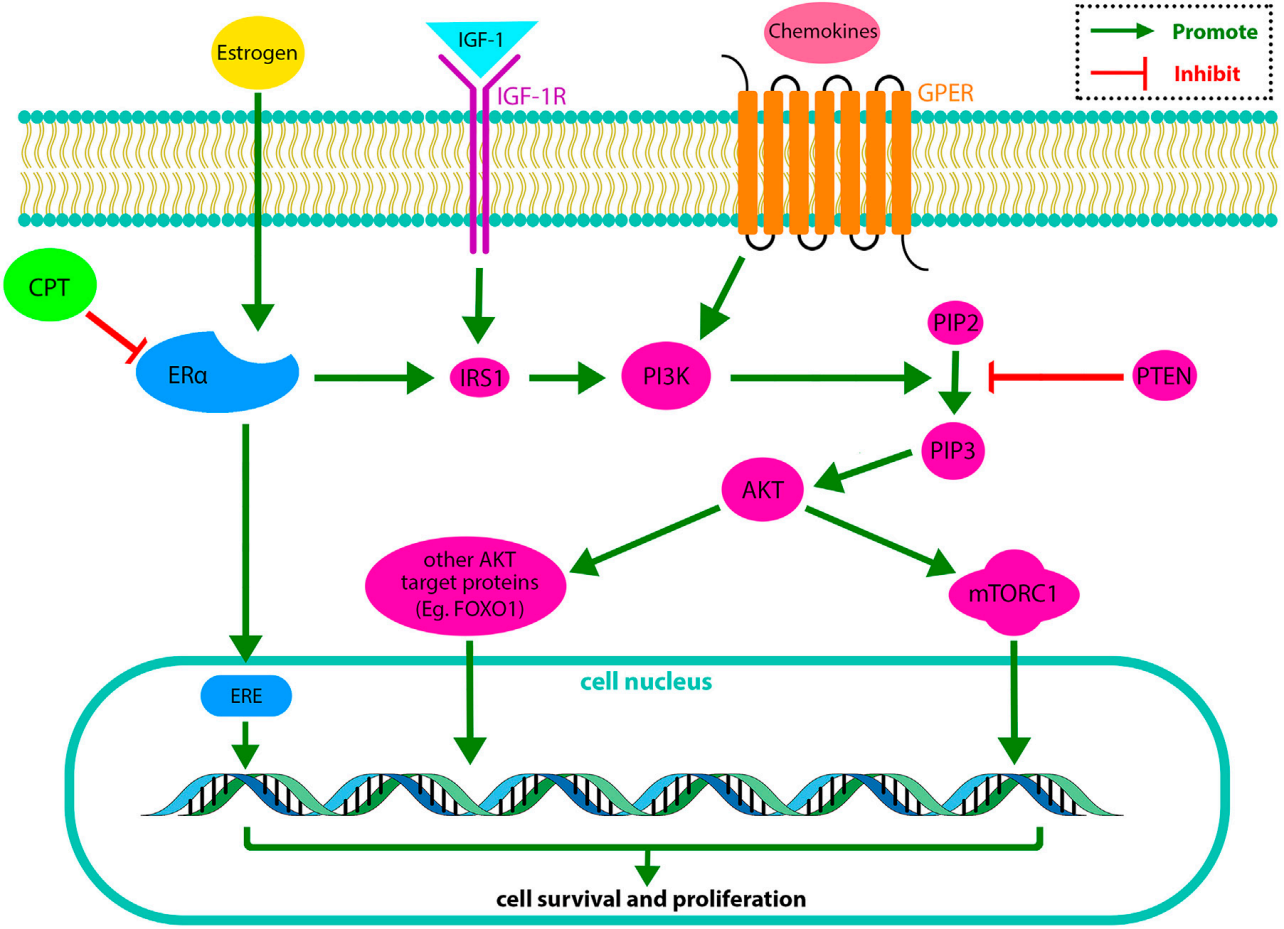
In the PI3K/AKT/mTOR pathway, IGF-1 activates IGF-1R, and some cytokines activate GPER in the cell membrane. IGF-1R and GPER activate PI3K. The activated PI3K has a catalytic effect on the phosphorylation of PIP2 to produce PIP3. PIP3 drives AKT. The activated AKT phosphorylates target proteins in the cytoplasmic fluid and cell nucleus. Finally, phosphorylation of AKT target proteins regulates cell survival and replication. On the other hand, PTEN (a PIP3 phosphatase) changes PIP3 to PIP2, suppressing the PI3K/AKT/mTOR pathway. The ERα is another receptor, activating PI3K through stimulating IRS1. CPT exerts its inhibitory effect on the PI3K/AKT/mTOR pathway by inhibiting ERα (Miricescu et al., 2020). **IGF-1**, insulin-like growth factor 1; **IGF-1R**, insulin-like growth factor receptor 1; **GPER**, G protein-coupled receptor; **IRS1**, Insulin receptor substrate 1; **PI3K**, Phosphatidylinositol 3-Kinase; **PIP2**, Phosphatidylinositol (4,5)-bisphosphate; **PIP3**, phosphatidylinositol (3,4,5)-trisphosphate; **PTEN**, phosphatase and tensin homolog; **AKT**, serine/threonine protein kinase; **mTORC**, mammalian Target of Rapamycin complex; **FOXO1**, Forkhead box other 1; **ERE**, estrogen-responsive element.

Pan et al. (2017) demonstrated that the CPT-ERα binding affinity is close to estrogen and roughly half of the Tamoxifen, presenting the anti-estrogen potential of CPT. Therefore, CPT could inhibit cell survival, growth, invasion, and migration of ERα-positive BRCA (MCF-7) cells via different mechanisms through competitive binding to ERα. CPT inhibited the ERα-mediated IGF-1/AKT/mTOR signaling and suppressed the IRS-1/AKT cascade. Thus, CPT inhibited the AKT-mTOR cascade in MCF-7 BRCA cells. Figure 1 demonstrates the regulatory effect of CPT on ERα-positive BRCA cell survival and proliferation.

Another study by Li et al. (2015) indicated that though CPT slightly downregulated ERα expression levels, CPT-ERα competitive binding is more pivotal than downregulating ERα protein expression. CPT could significantly inhibit the viability and proliferation of breast cancer cells due to the reduction of ERα target genes transcription *via* competitive binding to ERα protein. Thus, it has more efficacy in the treatment of ERα-positive rather than ERα-negative BRCA.

To find out other anti-cancer mechanisms of CPT, the differentially expressed genes (DEGs) were identified on both ERα-positive and -negative BRCA cell lines (Li et al., 2021). The results suggested three main DEGs. The Estrogen Receptor Gene (ESR1), the Cyclin-Dependent Kinase 1 (CDK1), and CCNA2. CPT intervention decreased the expression of CCNA2 and CDK1 in both cell lines, predominantly in ERα-positive BRCA cells, while no changes were observed in the ESR1 gene expression in either of the two cell lines (Pagano et al., 1992; Stein and Yang, 1995; Wang et al., 2011; Li et al., 2021).

Pharmacological interventions, which can induce prolonged endoplasmic reticulum stress (ER-stress), has been recently suggested as a possible method for tumor therapy (Healy et al., 2009). Park et al. (2012) realized that CPT induces ER-stress markers by generating reactive oxygen species (ROS). Further, the apoptosis biochemical markers (the phosphorylation level of eIF2a and protein levels of CHOP, GRP94, and GRP78), increase of sub-G1 DNA, and induction of DNA fragmentation were found in the MCF-7 cells exposed to CPT. All suggested that CPT as a natural compound induces ER-stress -mediated apoptosis in MCF-7 breast cancer cells.

Zhou et al. (2014) investigated the novel anti-tumor therapeutic role of CPT. They showed that CPT could perform its anticancer effect by stimulating the immune system, through CD4^+^ T cells by promoting secretion of IFN-γ or perforin. CPT acts like IL-12 and causes the release of perforin from CD4^+^ T cells through the phosphorylation of the JAK2/STAT4 pathway, mainly inhibited the growth of breast cancer cells.

### Cryptotanshinone against estrogen receptor alpha-negative breast cancer cells (MDA-MB 231, SKBR-3, Bcap37 cell lines)

ERα-negative breast cancer treatment is a big challenge due to its poor prognosis. Previous studies demonstrated the ERα-negative breast cancer resistance against anticancer drugs (Lappano et al., 2014; Bhat et al., 2015). Thus, finding an effective treatment for ERα-negative BRCA is crucial. Some subgroups of ERα-negative BRCA cells, such as SKBR-3 cells, are membrane G protein-coupled estrogen receptor (GPER) positive (Steiman et al., 2013). *In vitro* research claimed that GPER might function as a tumor suppressor in BRCA cells (Ariazi et al., 2010; Weißenborn et al., 2014). Recent studies revealed that GPER and its mediated signaling pathway [phosphatidylinositide 3-kinase (PI3K)/AKT] have a vital role in the proliferation of BRCA cells (Molina et al., 2017; Hsu et al., 2019).

It has been demonstrated that CPT treatment significantly downregulated the GPER-mediated PI3K/AKT signaling pathway of the ERα-negative human breast cancer cells, SKBR-3, in a dose and time-dependent manner. CPT might arrest the cell cycle associated with GPER-mediated G1-phase block. In addition, the expression of cyclin and CDK, which modulate the cell cycle regulation, obviously decreased after CPT treatment in a dose-dependent manner (Shi et al., 2020).

Bcap37 cells, as an ERα-negative BRCA cell line, have more migration and invasion than ERα-positive BRCA cells. CPT can potentially be an apoptosis inducer, anti-proliferative, and tumor-migration inhibitor drug in the ERα-negative BRCA cell lines. Liu et al. (2016) indicated that CPT could inhibit the proliferation and migration of Bcap37 cells and could induce apoptotic pathways in a dose- and time-dependent manner by arresting the cell cycle at the S phase during interphase. The main responsible for the cytotoxic effects of CPT in the ERα-negative BRCA cells is the inhibitory effect on FOXO1 (Thannickal and Fanburg, 2000; Akasaki et al., 2014).

### Cryptotanshinone in combination with other drugs

Breast cancer metastasis is a complex condition in which the tumor microenvironment plays an important role. Therefore, modulation of the tumor microenvironment through various biochemical pathways can have an anti-metastatic effect (Gao et al., 2009). Silibinin (SLB) is an herbal product that constrains tumor angiogenesis and reduces epithelial-mesenchymal transition (Deep and Agarwal, 2010; Deep and Agarwal, 2013). By modulating the tumor microenvironment via different pathways, SLB, as well as CPT, are known as anti-metastatic natural products.

Liu et al. (2020) assessed the bioavailability and anti-metastatic efficacy of oral nanoparticles for administrating the SLB and CPT lung metastasis in a 4T1 breast cancer tumor-bearing nude mouse model. They demonstrated that Silibinin- and cryptotanshinone-co-loaded nanoparticles (S/C-W-LPNs) significantly induced cell toxicity compared to SLB-co-loaded nanoparticles (S-W-LPNs) or CPT-co-loaded nanoparticles (C-W-LPNs) alone. Further, *in vitro* anti-metastasis study showed that S/C-W-LPNs markedly inhibited cell invasion and migration; with a relative cellular migration rate of 8.6% ± 1.38% which was less than those for C-W-LPNs and S-W-LPNs (15.5% ± 3.58%, 19.9% ± 3.35%, respectively).

Arsenic trioxide (As_2_O_3_) is known as a successful treatment for acute promyelocytic leukemia worldwide (Zhu et al., 1997). Zhang et al. (2015) explored a new therapeutic method for the treatment of ER-positive breast cancer. They exposed the MCF-7 BRCA cell line to three arsenic species, namely inorganic arsenite (iAs^III^), its intermediate metabolites monomethylarsonous acid (MMA^III^), and dimethylarsinous acid (DMA^III^) either alone or in combination with CPT and investigated their anti-breast cancer effects. The findings suggested that the combination of MMA^III^ with CPT has a remarkable synergic cytotoxic effect on cell viability. Further, they reported that MMA^III^ with CPT induces cellular apoptosis significantly (apoptosis rates up to 40%) compared to the combination of iAs^III^ or DMA^III^ with CPT, through changing the proapoptotic proteins Bax, Bak, and cyt c in the cytoplasm and mitochondria of BRCA cells (Zhang et al., 2015).

### Cryptotanshinone and conventional therapies

Recent significant progression in the cancerous cells’ drug resistance is a prominent obstacle for clinicians during chemotherapy. One of the solutions is using compounds that can synergize with conventional chemotherapy drugs. Park et al. (2012) evaluated the alone and the synchronic cytotoxic effects of CPT and chemotherapy drugs such as 5-FU, TNFα, etoposide, and cisplatin. They observed that lonely exposure to CPT or each antitumor drug has minimal effect on MCF-7 BRCA cells’ viability and has not any noticeable induction of ER-stress or apoptotic markers. On the other hand, synchronic use of CPT and antitumor drugs showed a prominent antitumor synergism with the promotion of apoptotic markers, indicating that CPT exerts its synergistic effect through potentiation of the apoptotic activity of different antitumor drugs *via* the stimulation of ER-stress (Park et al., 2012).

A study of treating C57 mice with cancerous MCF-7 cells with CPT or Taxol revealed that CPT remarkably inhibited the cancerous cells’ growth from day 13 compared to non-treatment mice. However, the therapeutic effect of CPT was minimally less than Taxol. But their findings suggested that CPT, along with conventional chemotherapy drugs, could have a synergic effect on breast cancer treatment (Zhou et al., 2014).

### Cryptotanshinone and multi-drug resistant breast cancer

Multi-drug resistance (MDR) in breast tumors is a condition that reduces the efficacy of chemotherapy drugs (Wang et al., 2017b). Often it occurs following long-term anti-estrogen chemotherapy and ERα-negative breast cancers. MDR has a tight association with breast cancer resistance protein (BCRP). BCRP is a membrane protein that causes efflux of chemotherapy drugs from tumor cells, therefore making cancer cells less affected by chemotherapy drugs (Mao and Unadkat, 2015; Li et al., 2016).

**FIGURE 2:**
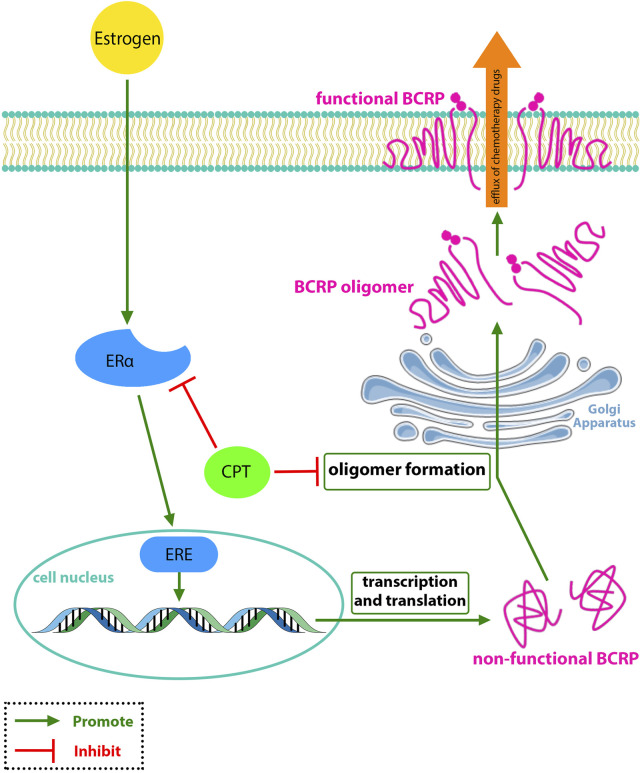
CPT interferes with the oligomer formation of BCRP and inhibits the BCRP function in the efflux of chemotherapy drugs which is associated with MDR-BRCA.

Thus, Ni et al. (2021) stated that BCRP might have a vital role in regulating the CPT transportation across the breast cancer cells membrane. They found that although CPT could not affect the intracellular protein and mRNA levels of BCRP/ABCG2, but inhibited the efflux function of BCRP in MCF-7 cells by reducing the BCRP expression on the cell membrane, which was ERα-dependent (Figure 2). BCRP is primarily of dimer and oligomer formation on the MCF-7 cell membrane. To find whether CPT was synergistic with BCRP-mediated efflux of anticancer drugs or not, they investigated the effect of CPT along with two of the most common BCRP drugs, MX and TOPO, compared with treatment with MX or TOPO alone. The findings showed that CPT increased the efficacy of chemotherapy drugs that can be effluxed by BCRP from tumor cells, reversing MDR (Ni et al., 2021).

In a similar study, Pan et al. (2017) used cancer cells with the acquired multidrug resistance (MCF-7/ADR). The results showed an undetected Tamoxifen cytotoxic effect on the MCF-7/ADR cancer cells, while CPT had a significant inhibitory effect. Moreover, CPT conjoined with Tamoxifen plies a synergic effect on the MCF-7/ADR cells. These findings indicated that CPT suppresses cell viability and proliferation in the tamoxifen-resistant BRCA cells especially combined with Tamoxifen.

### Cryptotanshinone inhibits the metabolism of breast cancer cells

Cancerous cells have a higher metabolic rate to promote tumor proliferation and progression (Fanciulli et al., 2000; Lu et al., 2008). Expression of glycolysis-related proteins, like HK2, LDHA, and PKM2, increases in breast cancer cells. PKM2, a poor prognostic marker, is an enzyme that induces glycolysis in breast cancer cells. CPT reduces the expression of PKM2 in both ERα-positive and ERα-negative breast cancer cells, inhibiting glycolysis. Glycolysis inhibition reduces metabolic rate and increases the sensitivity of cancerous cells to chemotherapy drugs (Zhou et al., 2020).

It is worth noting that some articles clarified that PKM2 also could be translocated into the nucleus of breast cancer cells, functioning as a transcription factor that transactivates β-catenin. β-Catenin is one of the most important mediators of angiogenesis, invasion, and cell migration in breast cancer (Yang et al., 2011; Wang et al., 2017a). Therefore CPT inhibits the invasion and migration of breast cancer cells by inhibiting the PKM2/β-catenin axis in both ERα-positive and -negative breast cancer cell lines (Zhou et al., 2020).

## Conclusion

Breast cancer incidence is dramatically increasing year by year in women worldwide. It has recently passed the other malignancies and has become the most life-threatening female cancer with the first rank (Sung et al., 2021). Despite various treatments available for different severity of breast cancer, including surgical resection, chemotherapy, and radiotherapy, there are types of BRCA with poor prognosis, particularly triple-negative breast cancer. While surgical resection is prescribed for only a minority of BRCA patients, most patients undergo chemotherapy which has increased the patients’ lifelong. A beneficial drug causes minimal damage to healthy body cells while having the most cytotoxicity to cancer cells. Therefore, suggesting new anticancer medicine with greater efficacy and fewer side effects is one of the hotspots for cancer treatment in recent years.

Cryptothanshinone, a derivation of the plant *S. miltiorrhiza* Bunge, has been recently considered by researchers due to its numerous anti-inflammatory and antitumor activity *in vivo* and *in vitro*. Notably, this Chinese herbal medicine is efficient in cancer treatment through different mechanisms, including targeting various molecular signaling pathways. In this study, we focused on the anti-cancer activity of CPT against breast cancer and summarized the various biological mechanisms through which CPT affects different types of BRCA. Most studies were conducted using ERα-positive and -negative BRCA. But no study considered the TNBC type. Therefore, evaluating the effects of CPT on the most challenging type of breast cancer, TNBC, is recommended for future studies. For ERα-Positive BRCA cells, studies have shown that CPT inhibits proliferation, migration, invasion, and cell viability of tumor cells *in vivo* or *in vitro*. Effects on estrogen receptor function, regulation of gene expression, endoplasmic reticulum stress-induced apoptosis, induction of the immune response, and inhibition of glycolysis are mechanisms that are affected by CPT.

In the case of ERα-negative BRCA cells, *in vivo* and *in vitro* studies have demonstrated that CPT provided its anti-cancer effect through GPER-mediated pathways, apoptosis *via* reactive oxygen species, and to a lesser extent through regulation of gene expression. Also, CPT has shown a synergic effect, more bioavailability, and more induced sensitivity to chemotherapy when combined with other drugs such as Silibinin, arsenic species, and conventional chemotherapy drugs. In summary, cryptotanshinone should be recognized as herbal medicine that offers many antitumor mechanisms and has considerable potential for treating female breast cancer.
